# Construction of a high-density linkage map and graphical representation of the arrangement of transcriptome-based unigene markers on the chromosomes of onion, *Allium cepa* L.

**DOI:** 10.1186/s12864-021-07803-y

**Published:** 2021-06-26

**Authors:** Satoshi Fujito, Turgut Yigit Akyol, Takuya Mukae, Tadayuki Wako, Ken-ichiro Yamashita, Hikaru Tsukazaki, Hideki Hirakawa, Keisuke Tanaka, Yoko Mine, Shusei Sato, Masayoshi Shigyo

**Affiliations:** 1 Institute of Vegetable and Floriculture Science, National Agriculture and Food Research Organization (NARO), 360 Kusawa, Ano, 514-2392 Tsu Mie, Japan; 2 Graduate School of Life Sciences, Tohoku University, 2-1-1 Katahira, 980-8577 Sendai, Miyagi Japan; 3 Department of Molecular Biology and Genetics, Aarhus University, Gustav Wieds Vej 10, DK-8000 Aarhus C, Denmark; 4 Graduate School of Sciences and Technology for Innovation, Yamaguchi University, 1677-1 Yoshida, 753-8515 Yamaguchi, Yamaguchi Japan; 5 Ministry of Agriculture, Forestry and Fisheries, 1-2-1 Kasumigaseki, Chiyoda- ku, 100-8950 Tokyo, Japan; 6 Western Region Agricultural Research Center, NARO, 1-3-1 Senyu-cho, 765-8508 Zentsuji-shi, Kagawa Japan; 7 Tohoku Agricultural Research Center, NARO, 4 Akahira, Shimo-kuriyagawa, 020-0198 Morioka, Iwate Japan; 8 Kazusa DNA Research Institute, 2-6-7 Kazusa-Kamatari, Kisarazu, 292-0818 Chiba Japan; 9 The NODAI Genome Research Center, Tokyo University of Agriculture, 1-1-1 Sakuragaoka, Setagaya-ku, 156-8502 Tokyo, Japan; 10 Department of Agriculture, Tokyo University of Agriculture, 1737 Funako, 243-0034 Atsugi-shi, Kanagawa Japan

**Keywords:** *Allium cepa* L., Bulb onion, Linkage map, Shallot, Transcriptome-based SNP genotyping

## Abstract

**Background:**

Genomic information for *Allium cepa* L. is limited as it is heterozygous and its genome is very large. To elucidate potential SNP markers obtained by NGS, we used a complete set of *A. fistulosum* L.-*A. cepa* monosomic addition lines (MALs) and doubled haploids (DHs). These were the parental lines of an *A. cepa* mapping population for transcriptome-based SNP genotyping.

**Results:**

We mapped the transcriptome sequence reads from a series of *A. fistulosum*-*A. cepa* MALs onto the unigene sequence of the doubled haploid shallot *A. cepa* Aggregatum group (DHA) and compared the MAL genotype call for parental bunching onion and shallot transcriptome mapping data. We identified SNP sites with at least four reads on 25,462 unigenes. They were anchored on eight *A. cepa* chromosomes. A single SNP site was identified on 3,278 unigenes and multiple SNPs were identified on 22,184 unigenes. The chromosome marker information was made public via the web database Allium TDB (http://alliumtdb.kazusa.or.jp/). To apply transcriptome based genotyping approach for genetic mapping, we gathered RNA sequence data from 96 lines of a DHA × doubled haploid bulb onion *A. cepa* common onion group (DHC) mapping population. After selecting co-dominant SNP sites, 16,872 SNPs were identified in 5,339 unigenes. Of these, at least two SNPs with identical genotypes were found in 1,435 unigenes. We developed a linkage map using genotype information from these unigenes. All unigene markers mapped onto the eight chromosomes and graphical genotyping was conducted based on the unigene order information. Another 2,963 unigenes were allocated onto the eight chromosomes. To confirm the accuracy of this transcriptome-based genetic linkage map, conventional PCR-based markers were used for linkage analysis. All SNP - and PCR-based markers were mapped onto the expected linkage groups and no inconsistency was found among these chromosomal locations.

**Conclusions:**

Effective transcriptome analysis with unique *Allium* resources successfully associated numerous chromosome markers with unigene information and a high-density *A. cepa* linkage map. The information on these unigene markers is valuable in genome sequencing and useful trait detection in *Allium*.

**Supplementary Information:**

The online version contains supplementary material available at 10.1186/s12864-021-07803-y.

## Background

The genus *Allium* comprises economically important vegetable crops such as bulb onion (*A. cepa* L.), garlic (*A. sativum* L.), bunching onion (*A. fistulosum* L.), leek (*A. porrum* L.), and numerous wild species (Hanelt, 1990) [1]. Bulb onion is a major vegetable crop worldwide. According to the FAOSTAT database, global bulb onion production was ~ 96 million t and ranked second after tomato in terms of vegetable crop cultivation in 2018 [2]. *Allium cepa* L. consists of the common onion (bulb onion) and the Aggregatum (shallot) groups (Jones and Mann, 1963) [3]. Shallot is also an important vegetable crop and is cultivated mainly in Europe, Southeast Asia, and Africa. Though it differs morphologically and ecologically from bulb onion, both are easily crossed (Astley et al., 1982) [4]. Shallot has a short growing period and is resistant to *Fusarium oxysporum* (Vu et al., 2012) [5]. Hence, analysis of its genome might generate valuable information applicable to bulb onion breeding. The latter is time-consuming and labor-intensive as bulb onion is a biennial and heterogeneous because of severe inbreeding depression. To facilitate bulb onion breeding efforts, then, it is necessary to develop effective methods such as DNA marker-assisted selection.

Various DNA markers have been developed for *Allium* species. Simple sequence repeat (SSR) markers have been used to construct linkage maps as they are co-dominant and useful for PCR-based detection (Baldwin et al., 2012; Bradeen and Havey, 1995; Ipek et al., 2005; Martin et al., 2005; McCallum et al., 2012; van Heusden et al., 2000a, 2000b; Wilkie et al., 1993; Tsukazaki et al., 2006, 2007, 2008, 2011, 2015; Fischer and Bachman, 2000; Song et al., 2004) [6–20]. However, only ~ 500 SSR markers have been developed for bulb onion so far (Fischer and Bachman, 2000; Kuhl et al., 2004; McCallum et al., 2006; Tsukazaki et al., 2008, 2011; Martin et al., 2005) [9, 16, 17, 19, 21, 22]. These are inadequate for precise genetic analysis and DNA marker-assisted selection in bulb onions.

The advent of next-generation sequencing (NGS) has realized the accumulation of large amounts of sequence information and the identification of numerous single-nucleotide-polymorphisms (SNPs) to develop markers in plants with large genomes (Takahagi et al., 2016) [23]. NGS has been used to generate SNP markers in bulb onions via transcriptomic and selected genomic regions (Duangjit et al., 2013; Jo et al., 2017; Choi et al., 2020) [24–26]. Numerous SNPs were identified by these approaches. However, only 597, 202, and 319 SNP markers were anchored on each genetic map, respectively, because of parental line heterozygosity in the mapping population and a lack of reliable reference genome sequences. For the effective use of NGS technology for plants without reference genome sequences, plant materials with sufficient homozygosity must be applied to the parental lines of the mapping population.

Doubled haploid (DH) techniques use chromosome doubling of haploid plants to generate materials that are homozygous enough for genetic analysis (Alan et al.,2003; Ajisaka et al., 2001; Jia et al., 2005) [27–29]. For *Allium*, we developed shallot and bulb onion DH lines and their F_1_ hybrids for use in genetic analysis (Abdelrahman et al., 2015; Wako, 2016) [30, 31]. We also developed several bunching onion (*Allium fistulosum* L.)-shallot monosomic addition lines (MALs) (Shigyo et al., 1996) [32]. These have been used to assign genetic linkage maps to *A. cepa* chromosomes by seeking shallot-type alleles among the eight MALs (van Heusden et al., 2000b; Martin et al., 2005) [9, 12]. The combination of these plant resources could enhance potential SNP genotyping by NGS.

Here, we performed a transcriptome analysis on MALs to generate information about chromosome-specific unigene markers. We conducted transcriptomics on the F_2_ population derived from a cross between shallot and bulb onion DH lines. We also constructed a high-density genetic linkage map by elucidating the potential SNP sites generated by NGS.

## Results and discussion

### Unigene chromosome anchoring by SNP genotyping via MAL RNA sequencing

In our previous study, we carried out transcriptome analysis of a complete set of MALs using RNA samples isolated from the leaf, root, and bulb (Abdelrahman et al., 2017) [33]. In the evaluation process of expression level, we obtained transcriptome reads of each MAL, and parental bunching onion and doubled haploid shallot (DHA) were mapped onto unigene data set constructed using DHA bulb transcriptome as a reference. During this analysis, we realized that SNPs between bunching onion and DHA could be identified in the mapping data, and these SNPs could be applied for anchoring the unigene sequences onto shallot chromosomes. Theoretically, in genotype calling, MALs having the shallot chromosome on which the target gene is allocated become heterozygous, whereas the remaining MALs become homozygous of bunching onion allele. Therefore, we performed transcriptome based genotyping using previously accumulated data (Abdelrahman et al., 2017) [33], as advanced mapping data applications. SNP sites with alternative homozygous calls in bunching onion and reference homozygous calls in DHA were selected by comparing the genotype call of the transcriptome mapping data between the MAL parental lines (bunching onion and shallot). Among 56,161 DHA unigenes, sites with ≥ 4 reads coverage in all eight MALs were identified on 25,462 unigenes (Table 1). Of these, one SNP was identified in 3,278 unigenes, which could be anchored onto a chromosome as a heterozygous genotype were called one MAL. On the contrary, multiple SNP sites were identified in 22,184 unigenes. Of these, 21,996 could be allocated to single physical chromosomes. Extrachromosomal MALs with heterozygous genotypes are consistent with chromosomal unigene locations with multiple SNPs. For the remaining 188 unigenes, ≥ 2 multiple SNPs were ambiguous. There were heterozygous genotypes in eight MAL types and/or parental homozygous genotype(s). The corresponding gene may have been downregulated and the shallot gene had partial homology. These unigenes were assigned to the chromosome based on other marker(s) with a single heterozygous genotype in MALs with the “R” indication and mapped by representative SNPs. A total of 25,462 unigenes were anchored on eight chromosomes. There were 4,513 unigenes on chromosome 2 and only 2,169 unigenes on chromosome 8.

**Table 1:** DHA unigenes with SNP(s) detected on MALs

		Chr. 1	Chr. 2	Chr. 3	Chr. 4	Chr. 5	Chr. 6	Chr. 7	Chr. 8	Total
Multiple SNPs	allocated to single chromosome	3,404	3,900	3,148	2,538	2,588	2,546	2,032	1,840	21,996
	including representative SNP	30	35	33	21	16	16	18	19	188
One SNP		486	578	455	397	357	376	319	310	3,278
Total		3920	4,513	3,636	2,956	2,961	2,938	2,369	2,169	25,462

DHA unigene information has been made public through the web database ‘Allium Transcriptome DataBase’ (TDB) at http://alliumtdb.kazusa.or.jp. We have added the list of chromosome markers in this data base (Table S1). These anchoring markers are useful in genome sequencing projects.

### SNP detection in ***Allium cepa*** doubled haploids

To expand the transcriptome based genotyping approach for the construction of genetic linkage map and genetic markers, we accumulated transcriptome data of the F_2_ plants derived from a cross between the *A. cepa* DH lines (DHA for shallot × DHC for bulb onion). RNA sequence of leaf samples was collected from 96 F_2_ plants (population A) of the mapping population and from DHC. Using the Illumina sequencing platform, 10–25 million 100 base paired end sequence reads were accumulated for each plant, and the obtained reads were mapped onto DHA bulb unigene data set used in the MAL transcriptome analysis. As the parental lines were doubled haploid, genotyping of the F_2_ plants from the mapping population should be classified as reference (DHA) homozygous, alternative (DHC) homozygous, and heterozygous. The intraspecific SNPs identified by mapping DHC reads with ≥ 2 reads coverage on all 96 lines were selected for genotyping. Selecting co-dominant SNP sites with heterozygous genotypes among the 96 lines identified 16,872 SNP sites in 5,339 unigenes. One SNP site was identified on 2,109 unigenes. These genotypes were used for map calculation with an “O” indication meaning that one SNP site was supported. Of the 3,230 unigenes with multiple SNP sites, ≥ 2 SNP sites with identical genotype patterns on the 96 lines were identified on 1,435 unigenes. These patterns were selected as the solid genotype (S) of the corresponding unigenes. For the remaining 1,795 unigenes, inconsistencies between the homozygous and heterozygous calls were identified among the 96 lines. The representative genotype (R) was created by selecting the most abundant genotype in each of the 96 lines.

### Genetic linkage map construction and physical chromosome assignment

We used the solid co-dominant genotype information obtained from 1,435 unigenes in population A to plot a genetic linkage map with JoinMap v. 4.0 (Kyazma BV, Wageningen, The Netherlands). By applying the LOD 5 cutoff, all tested markers were assigned to eight linkage groups. Based on the unigenes with anchored chromosome information, all of these could be anchored to each of the eight bulb onion chromosomes. No inconsistency was detected between each linkage group and assigned chromosome. Hence, this linkage map was reliable.

A graphical genotype list was constructed according to the unigene order information. A total of 610 genotype blocks were assigned based on the patterns of the tested 96 lines (Table S2). The remaining unigenes with “O”- and “R”-coded genotypes were allocated to the most probable genotype block and permitted genotype inconsistencies for ≤ 10 lines. A total of 1,537 “O”-marked and 1,426 “R”-marked unigenes were allocated onto the genotype blocks (Table 2, Supplemental file). As the inconsistency of genotyping in R-marked tends to be a homozygous call in heterozygous allele, lower expression level and/or allele bias in expression could be potential sources of errors in transcriptome based genotyping.

**Table 2:** Genetic markers developed for each chromosome

	Chr. 1	Chr. 2	Chr. 3	Chr. 4	Chr. 5	Chr. 6	Chr. 7	Chr. 8	Total
Solid (S)	270	126	200	170	199	198	170	102	1,435
Representative(R)	175	416	195	163	148	123	110	96	1,426
One (O)	239	278	229	192	135	186	148	130	1,537
Total	684	820	624	525	482	507	428	328	4,398

**Fig. 1:**
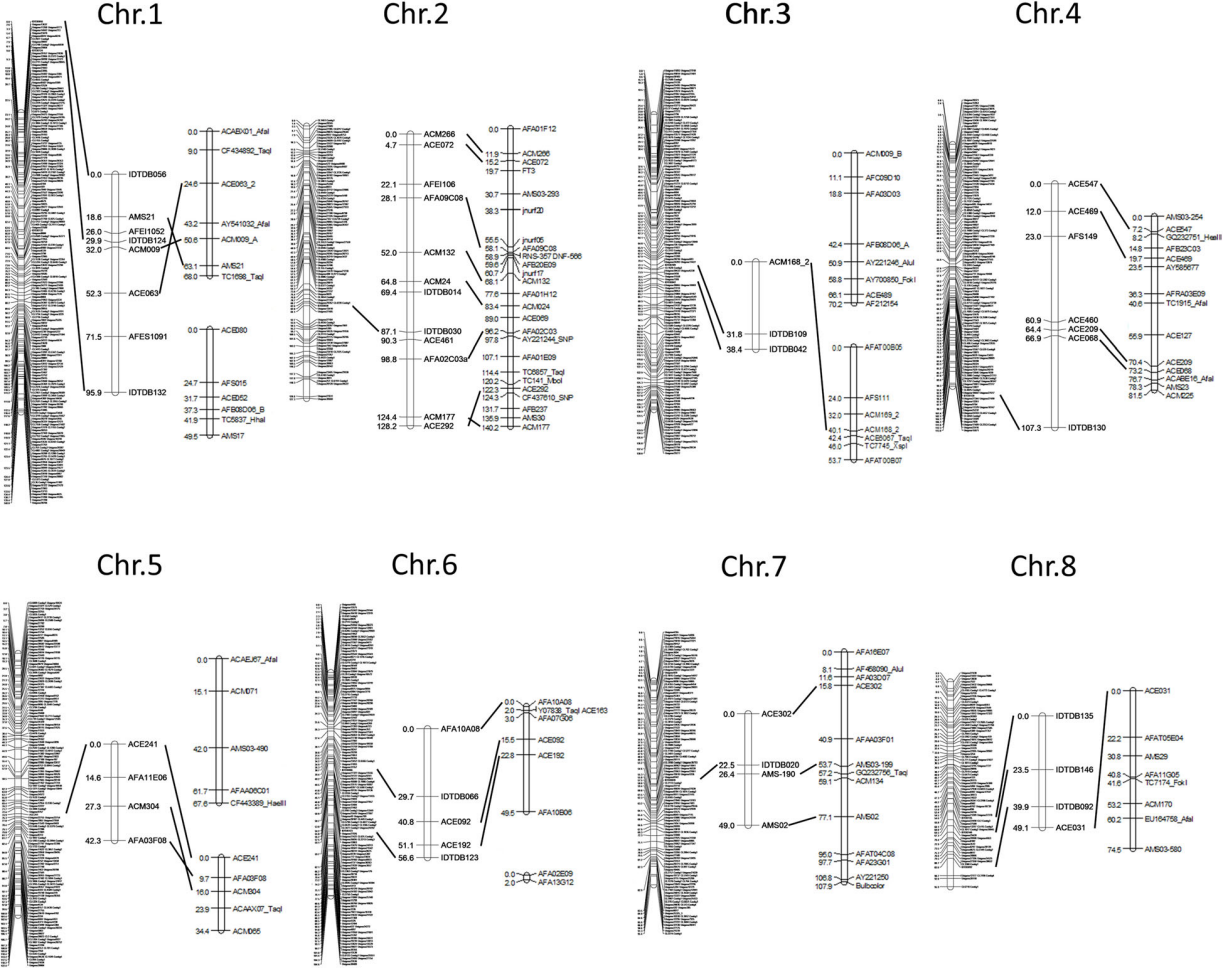
Linkage maps. Left, SNP-based map including PCR-based markers (F_2_ population **A**). Middle, PCR-based map (F_2_ population **B**). Right, linkage map constructed using a gynogenic population derived from F_1_ (population **C**) (Wako, 2016)

To confirm transcriptome-based genetic linkage map accuracy, we applied conventional PCR-based markers to the same F_2_ population (A). The PCR-based SSR and InDel markers were previously reported (Fischer and Bachmann, 2000; Kuhl et al., 2004; Martin et al., 2005; McCallum et al., 2012; Tsukazaki et al., 2006, 2007, 2008, 2011; Wako, 2016) [9, 10, 14–17, 19, 21, 31] and used in the present study. Thirty-three markers were polymorphic between DHA and DHC. Fourteen InDel polymorphisms were detected for the sequence comparisons between DHA and DHC in Allium TDB. We designed primer sets that included these polymorphism sites and amplified them by PCR. We used 47 PCR-based markers in a linkage analysis on another F_2_ population (B). All linkage groups were assigned to eight physical chromosomes in MALs confirmed by amplification. These marker locations matched those in previous reports (Tsukazaki et al., 2008, 2011, 2015; Masuzaki et al., 2006a, 2006b; Wako, 2016) [16–18, 31, 34, 35]. We selected 14 reliable PCR-based markers covering all eight chromosomes, applied them to population A, and integrated them onto the transcriptome-based genetic linkage map. The reconstructed map consisted of eight linkage groups with all SNP solid and PCR-based markers covering 936.6 cM. The average marker interval was 0.65 cM. All PCR-based markers were integrated onto positions corresponding to those on population B. The latter was based on a PCR marker-based linkage map. No contradiction in marker location was caused by using common markers between these maps and another linkage map previously constructed with a gynogenic population (C) derived from the same F_1_ hybrid between DHA and DHC with the exception of some on Chr. 2 and Chr. 5 (Fig. 1) (Wako, 2016) [31]. We also compared the genetic maps against a published transcriptome-based SNP marker analysis (Duangjit et al., 2013) [24]. Comparison of the positions of 137 SNP markers on sequences overlapping in both analyses revealed that the anchored chromosomes and relative positions were consistent for all SNP markers (Table S3). Therefore, our transcriptome-based genetic linkage map is reliable.

MALs have been used extensively to assign DNA markers to physical chromosomes (van Heusden et al., 2000b; Martin et al., 2005; Tsukazaki et al., 2008) [9, 12, 16]. Here, we identified chromosome-specific SNPs by comparing transcriptome data with MALs. For the first time, we used F_2_ populations from *Allium* DH parental lines. The parental line has each homozygous allele. SNPs between the parental lines DHA and DHC are easily detected. Transcriptome data from the DH lines efficiently found SNPs (Baldwin et al., 2012) [6] and we obtained abundant and reliable SNP information here. We constructed a reliable genetic map based on S-marked SNP markers. No inconsistency was found between the physical chromosome assignments and S-labeled markers in the linkage group. The genetic map comprised 1,435 SNP markers, one bulb onion SSR marker, and 13 InDel markers and covered 936.6 cM. To our knowledge, this map has the highest number of markers to date. Integrated linkage maps include markers associated with phenotypic characteristics for the nuclear male fertility restoration loci of cytoplasmic male sterility (Chr. 2) and bulb color (Chr. 7) (Wako, 2016) [31]. Shallot is a genetic breeding resource for bulb onion as it produces certain distinctive chemical compounds such as saponins conferring pathogen resistance (Shigyo et al., 1997; Abdelrahman et al., 2017) [33, 36]. By combining these DH lines with linkage map information, progress is anticipated in *Allium* molecular breeding by marker-assisted selection for several agronomic bulb onion traits.

## Conclusions

In the present study, we constructed a high-density linkage map in *Allium cepa* using numerous SNP markers obtained from the transcriptome information of the *Allium* DH lines and the MALs. As DH techniques depress inbreeding, they are useful for making homozygous pure lines that resemble inbred lines (Abdelrahman et al., 2015) [30]. Though bulb onion and shallot have different characteristics, they both belong to *A. cepa* and are easy to cross (Astley et al., 1982) [4]. The MALs have all *A. fistulosum* chromosomes and one *A. cepa* chromosome (Shigyo et al., 1996) [32]. We performed a transcriptome analysis to identify unigenes and assign them to physical chromosomes. To this end, we compared shallot DH and MAL transcriptome data. We then used the F_2_ mapping population between bulb onion DH and shallot DH to detect SNP sites. A total of 16,872 SNP sites were identified on 5,339 unigenes. Of these, 1,435 were selected as the solid genotype of the corresponding unigenes. By constructing a linkage map with SNP solid markers, all markers were mapped and the locations between the physical chromosomes and linkage groups were consistent. The number of SNPs located on the linkage map was much higher than those previously reported. Thus, the linkage map resolution was high. Furthermore, linkage maps integrated with PCR-based markers are now available. Shallots produce chemical compounds conferring resistance to certain bulb onion diseases (Abdelrahman et al., 2017) [33]. Hence, connecting phenotype and genotype information is a holistic approach towards *Allium* gene expression analysis for plant breeding and an effective, low-cost method of developing novel disease-resistant *Allium* varieties.

## Methods

### Plant materials and genetic cross

F_2_ plants generated by a cross between shallot and bulb onion doubled haploid (DH) lines (DHA and DHC, respectively) were used to construct a linkage map. DHA was derived from ‘Chiang Mai’ shallot in Thailand while DHC was derived from long-day ‘Sapporo-ki’ onion cultivar in Japan (Abdelrahman et al., 2015) [30]. For the transcriptome and conventional PCR-based marker analyses, populations A (96 individuals) and B (186 individuals) were created. They were raised in the greenhouses of Yamaguchi University (34°11” N; 131°28” E) and the Institute of Vegetable and Floriculture Science of NARO (34°61” N; 136°25” E) in Japan.

For the *A. cepa* unigene chromosome assignments via interspecific SNP detection, a complete set of MALs of *Allium fistulosum* L. with eight single shallot extra-chromosomes (Shigyo et al., 1996) [32] was used (Abdelrahman et al., 2017) [33].

### Transcriptome sequencing

The strategy for the accumulation of RNA sequencing data of MALs and *A. fistulosum* and construction of DHA bulb unigene data set has been described previously (Abdelrahman et al., 2017) [33].

For RNA sequencing of 96 F_2_ plants and DHC, total RNA was isolated from leaf samples using the RNeasy plant mini kit (QIAGEN Sciences, Germantown, MD, USA). RNA quality was checked with an Agilent 2100 Bioanalyzer (Agilent Technologies, Palo Alto, CA, USA). Samples with RNA integrity number (RIN) > 8.0 were selected for further use. The cDNA library was generated with a TruSeq™ RNA sample preparation kit (Illumina, San Diego, CA, USA) in accordance with the manufacturer’s instructions. Sequencing was performed on the Illumina HiSeq 2500 platform (Illumina, San Diego, CA, USA).

### SNP detection and selection

RNA sequence reads were filtered with PRINSEQ v. 0.20.4 (Schmieder and Edwards, 2011) [37] and fastx_clipper in FASTX-toolkit (http://hannonlab.cshl.edu/fastx_toolkit/). The filtered single-end reads were mapped onto DHA unigene sequences in end-to-end mode with Bowtie v. 22.1.0 (Langmead et al., 2009) [38]. The sequence alignment/map (SAM) format files were converted into BAM format with SAMtools v. 0.1.19 (Li et al., 2009) [39]. The BAM files were subjected to SNP calling with the mpileup option of SAMtools31 v. 0.1.19 and the mpileup2snp option of VarScan v. 2.3 to obtain a variant call format (VCF) file containing the SNP information.

### PCR-based marker analysis

Total DNA was prepared from individual plants according to the method of Song et al. (2004) [20]. To detect polymorphisms between DHA and DHC, SSR markers derived from the *A. cepa* genome (Fischer and Bachmann, 2000) [19], the *A. fistulosum* genome (Ohara et al., 2005; Song et al., 2004; Martin et al., 2005; Tsukazaki et al., 2006, 2007, 2008, 2011; Wako, 2016) [9, 14–17, 20, 31, 40], and *A. cepa* expressed sequence tags (ESTs) (Jakse et al., 2005; Kuhl et al., 2004; Martin et al., 2005; Tsukazaki et al., 2008; 2011) [9, 16, 17, 21, 41] and other CAPS and SCAR markers (Kuhl et al., 2004; Masuzaki et al., 2006a, 2006b; McCallum et al., 2006; Yaguchi et al., 2008) [21, 22, 34, 35, 42] were screened. Moreover, polymorphisms were detected between DHA and DHC nucleotide sequences obtained from transcriptome information in Allium Transcriptome DataBase (AlliumTDB) (Abdelrahman et al., 2017) [33] by sequence comparison with BLASTn. InDels > 6 bp were selected and primer sets including the polymorphism site were designed with Primer 3 (http://bioinfo.ut.ee/primer3/) (IDTDBxxx). The markers were applied to the F_2_ population and parental lines. PCR was performed in a 10-µL reaction mixture containing 10 ng template DNA, 0.2 µM of each primer, 0.2 mM of each dNTP, and 5 µL GoTaq Master Mix (Promega Corp., Madison, WI, USA). Amplification was performed for 35 cycles after initial denaturation at 94 °C for 4 min. Each cycle consisted of 15 s at 94 °C, 30 s at 55 °C, 1 min at 72 °C, and 4 min at 72 °C. The PCR products were evaluated by 2 % (w/v) agarose gel electrophoresis. Certain forward primers were fluorescently labeled with 6-FAM, NED, PET, or VIC dyes (Applied Biosystems, Foster City, CA, USA) before PCR. The PCR products were loaded onto a capillary DNA sequencer (ABI3730; Applied Biosystems, Foster City, CA, USA) and analyzed with GeneMapper v. 3.0 (Applied Biosystems, Foster City, CA, USA).

### ***Allium cepa*** linkage map construction by two methods and map comparison

Linkage analysis was performed with JoinMap v. 4.0 (van Ooijen, 2006) [43]. The Kosambi function was used to obtain the cM values (Kosambi, 1944) [44]. The DNA markers used to construct the *A. cepa* linkage map are listed in Table S4. Eight different *A. fistulosum*-*A. cepa* MALs were used to determine the corresponding physical chromosomes for the linkage groups. The linkage maps were compared according to the SNP and PCR-based markers by using the 14 anchor markers on both maps.

## Supplementary Information

**Additional file 1.**

**Additional file 2.**

**Additional file 3.**

**Additional file 4.**

**Additional file 5.**

## Data Availability

RNA-Seq data for the MALs and F_2_ mapping population (DHA × DHC) are available in the DDBJ sequence read archive under Accessions No. DRA005096 and DRA009194, respectively. The DHA unigene information has been made public through the web database ‘Allium Transcriptome DataBase’ (AlliumTDB) located at http://alliumtdb.kazusa.or.jp.

## Abbreviations

DH: Doubled haploid
NGS: Next-generation sequencing
PCR: Polymerase chain reaction
MAL: Monosomic addition lines
SSR: Simple sequence repeat
InDel: Insertion-deletion
EST: Expressed sequence tag
SNP: Single-nucleotide polymorphism
CAPS: Cleaved amplified polymorphic sequence
SCAR: Sequence characterized amplified region
